# Delineation of Interfaces on Human Alpha-Defensins Critical for Human Adenovirus and Human Papillomavirus Inhibition

**DOI:** 10.1371/journal.ppat.1004360

**Published:** 2014-09-04

**Authors:** Victoria R. Tenge, Anshu P. Gounder, Mayim E. Wiens, Wuyuan Lu, Jason G. Smith

**Affiliations:** 1 Department of Microbiology, University of Washington, Seattle, Washington, United States of America; 2 Institute of Human Virology and Department of Biochemistry and Molecular Biology, University of Maryland School of Medicine, Baltimore, Maryland, United States of America; University of Pennsylvania Medical School, United States of America

## Abstract

Human α-defensins are potent anti-microbial peptides with the ability to neutralize bacterial and viral targets. Single alanine mutagenesis has been used to identify determinants of anti-bacterial activity and binding to bacterial proteins such as anthrax lethal factor. Similar analyses of α-defensin interactions with non-enveloped viruses are limited. We used a comprehensive set of human α-defensin 5 (HD5) and human neutrophil peptide 1 (HNP1) alanine scan mutants in a combination of binding and neutralization assays with human adenovirus (AdV) and human papillomavirus (HPV). We have identified a core of critical hydrophobic residues that are common determinants for all of the virus-defensin interactions that were analyzed, while specificity in viral recognition is conferred by specific surface-exposed charged residues. The hydrophobic residues serve multiple roles in maintaining the tertiary and quaternary structure of the defensins as well as forming an interface for virus binding. Many of the important solvent-exposed residues of HD5 group together to form a critical surface. However, a single discrete binding face was not identified for HNP1. In lieu of whole AdV, we used a recombinant capsid subunit comprised of penton base and fiber in quantitative binding studies and determined that the anti-viral potency of HD5 was a function of stoichiometry rather than affinity. Our studies support a mechanism in which α-defensins depend on hydrophobic and charge-charge interactions to bind at high copy number to these non-enveloped viruses to neutralize infection and provide insight into properties that guide α-defensin anti-viral activity.

## Author Summary

Human α-defensins are an important component of the innate immune response and provide an initial block against a broad number of infectious agents, including viruses and bacteria. Characteristics of α-defensins that are necessary for their anti-bacterial activity have been identified, but our understanding of determinants required for activity against non-enveloped viruses is limited. In this work, we utilized alanine scan mutagenesis to systematically and comprehensively investigate the role of hydrophobic and charged residues of two α-defensins in binding to and/or neutralization of human adenovirus and human papillomavirus. Our results implicate common core hydrophobic residues as critical for inhibition of these non-enveloped viruses by the two α-defensins, with specificity provided by charged residues unique to each interaction. We also found that the number of α-defensin molecules bound to the virus was a stronger correlate of the anti-viral potency of the α-defensin mutants than their absolute affinity for the viral capsid. Understanding common characteristics of α-defensins important for non-enveloped virus binding will inform rules that govern the function of these abundant and multifaceted peptides in host defense.

## Introduction

Human α- and β-defensins are small (3–5 kDa), cationic peptides of the innate immune system with broad anti-microbial activity [1]. The six human α-defensins can be further divided by expression pattern and gene structure into myeloid [human neutrophil peptides (HNPs) 1–4] or enteric [human α-defensins (HDs) 5 and 6] classes [2], [3]. Despite their variable sequences, α-defensins share common structural features including a triple-stranded β-sheet fold, three intramolecular disulfide bonds, and a salt bridge [4]. The activity of α-defensins against both gram-negative and gram-positive bacterial pathogens has been well characterized, while their anti-viral properties are less well understood [4], [5]. Their capacity to neutralize enveloped viruses can be explained in part through properties identified in anti-bacterial studies, including lipid perturbation and their ability to function as lectins, although other mechanisms have been proposed [5], [6]. In contrast, these properties are insufficient to explain their ability to inhibit multiple non-enveloped viruses. In this regard, we have shown that HD5 neutralizes human AdV by binding to fiber and penton base proteins at the vertices of the icosahedral capsid, thereby stabilizing the capsid and preventing uncoating and subsequent genome exposure [7]–[10]. Similarly, recent studies have identified post-entry blocks of HPV and JC polyomavirus infection by HD5 [11], [12], suggesting that common mechanisms may govern α-defensin neutralization of non-enveloped viruses.

Extensive structure-function studies of multiple α-defensins have identified features that dictate their anti-bacterial activity, including a prominent role for dimerization and higher order multimerization [13]–[15]. Dimerization also contributes to α-defensin binding to glycoproteins and bacterial toxins [13], [15]. Equivalent structure-function studies of α-defensin anti-viral activity, particularly for non-enveloped viruses, are lacking. In a recent study, we identified certain arginine residues and the need for stable dimer formation as crucial for HD5 inhibition of AdV and HPV [16]. To more globally assess HD5 function and define a viral binding interface, we tested HD5 analogues from a comprehensive alanine scan library for their ability to neutralize AdV and HPV and for their binding kinetics to AdV capsid proteins. We define a critical patch on the surface of HD5 important for both HPV and AdV inhibition. We also show that the stoichiometry rather than affinity of HD5 binding to the AdV vertex correlates with anti-viral activity. Additionally, we identify regions important for HNP1 anti-AdV activity. Comparison of similarities and dissimilarities between these two α-defensins may inform general rules for α-defensins in innate anti-viral immunity.

## Results

### Hydrophobic residues are important for the anti-viral activity of HD5

**Figure 1:**
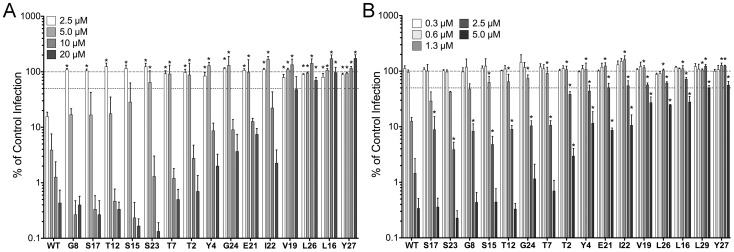
Anti-viral activity of HD5 and HD5 alanine scan mutants. Infection of A549 cells by AdV5.eGFP (*A*) or of HeLa cells by HPV16 PsVs encapsidating a GFP reporter plasmid (*B*) pre-bound to the indicated concentrations of α-defensins is expressed relative to control cells infected in the absence of α-defensin (100%, upper dashed line). Alanine was substituted for HD5 residues that are indicated by position and single letter code. Lower dashed line represents 50% of control infection. Data are the means of at least three independent experiments ± SD. *, p<0.05.

We have shown that specific arginine residues and the hydrophobicity of residue 29 are important for HD5 to function as an anti-viral molecule against human AdV and HPV [16]. Although human AdVs cause a broad spectrum of diseases, many serotypes are transmitted by the fecal-oral route, where they may encounter intestinal HD5 [9], [17], [18]. Similarly, HPV16 and related mucosal serotypes encounter HD5 in the female reproductive tract [19]. To systematically identify additional critical residues, we measured the effect of alanine substitutions in HD5 on its ability to neutralize a human AdV-5-based vector (AdV5.eGFP) and HPV16 pseudoviruses (PsVs). The anti-bacterial activity of these HD5 analogues has been reported [13]. Together with the single arginine substitutions from our previous study, they complete a collection of alanine substitutions for all non-alanine residues in HD5 except for those involved in three conserved features: a glycine (G18) required to form a β-bulge, a salt bridge (R6 and E14), and the six disulfide-bonded cysteine residues [13], [20], [21]. Briefly, defensins were incubated with AdV5.eGFP or HPV16 PsV, the mixtures were added to cells, and expression of GFP was quantified relative to control cells infected without defensin. HD5 was used at low micromolar concentrations, which are within the physiologic range of HD5 expression in the small intestine and female reproductive tract [5]. As expected, alanine substitutions for most of the residues in HD5 had less than a 2-fold effect on anti-viral activity (Figure 1). Substitution for hydrophobic residues or a bulky aromatic residue (Y27) had the greatest effect. The only exceptions were I22 for AdV and Y4 for both AdV and HPV. All of the residues that impacted AdV inhibition were also important for HPV, although in general there was less attenuation of HPV inhibition. When ranked by relative effect, the overall order of the alanine substitutions was consistent between the two viruses with the exception of E21, which was more important for HPV than AdV inhibition.

### Residues critical for anti-viral activity are located on one face of the HD5 dimer

**Figure 2:**
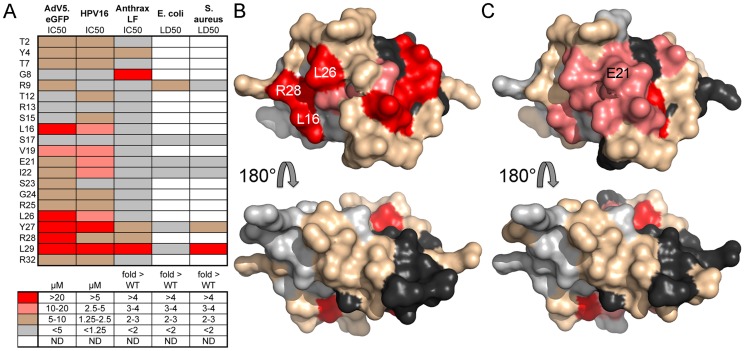
Residues critical for anti-viral activity are clustered on one face of HD5. *A*, heat map of the effect of alanine substitutions on the IC_50_ of HD5 against AdV5.eGFP (from Figure 1A and [16]), HPV16 (from Figure 1B and [16]), and anthrax lethal factor (LF, from [13]) or on the LD_50_ of HD5 against *E. coli* and *S. aureus* (from [13]) according to the color key below. ND = not done. *B,C*, surface rendering of the HD5 dimer (PDB: 1zmp) with residues colored as in *A* according to their effect on the IC_50_ of HD5 against AdV5.eGFP (*B*) or HPV16 (*C*). Black and light grey are equivalent and used to distinguish the HD5 monomers comprising the dimer. Molecular images were created with the PyMOL Molecular Graphics System (Schrödinger, LLC).

We used a heat map to collate our data from both studies and to compare the importance of HD5 residues for anti-viral and anti-bacterial activity (Figure 2A). Several residues critical for anti-viral activity are grouped towards the C-terminus of the peptide and are primarily hydrophobic, with the exception of the positively charged R28 [16]; however, L16 and V19 in the middle of the peptide sequence are also important for both AdV and HPV16 inhibition. With the exception of L29, residues critical for neutralization of viral infection differ from those required to kill bacteria [13], suggesting a distinctive mode of interaction between HD5 and these non-enveloped viruses.

As the critical anti-viral residues were separated in the primary structure, we visualized their organization on a space-filling model of the HD5 dimer (Figures 2B and C). The residues needed for neutralization of both AdV and HPV16 localize to one face of the dimer; whereas, mutation of residues on the opposite face had little or no effect. Key residues for inhibition of both AdV and HPV (L16, V19, and L26) are clustered together and surface exposed, while the side chains of L29 and Y27 are buried. For AdV, this hydrophobic surface is divided into two discrete patches and extended by the surface-exposed side chain of the positively charged R28. For HPV, inclusion of E21 forms a contiguous surface across the dimer interface.

### Creation of recombinant Penton

**Figure 3:**
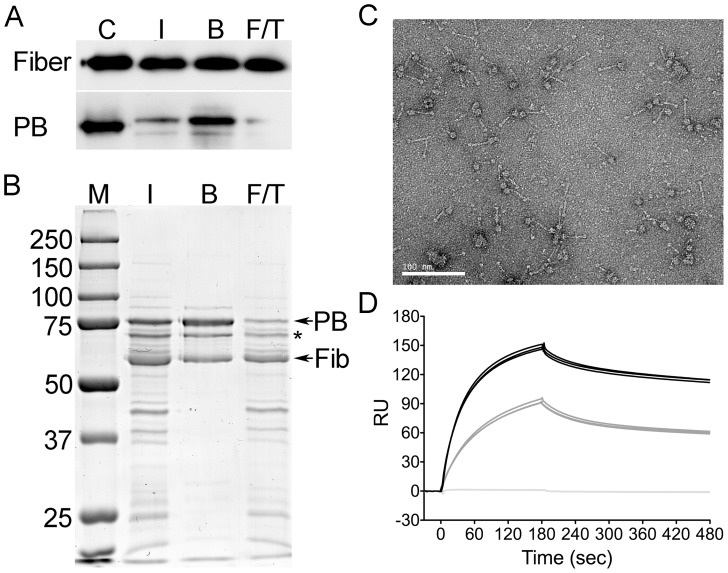
Purification and characterization of rPenton. *A*, immunoblots of input (I), bound (B), and flow-through (F/T) fractions from a representative rPenton purification were probed for fiber (top) or penton base (PB, bottom). Viral proteins were included as positive controls (C). *B*, fractions from this rPenton purification were separated by SDS-PAGE and stained with coomassie dye. Sizes of molecular weight standards (M) are indicated. * indicates a prominent non-viral protein impurity. *C*, electron micrographs of the resulting purified rPenton. Scale bar is 100 nm. *D*, background-corrected SPR sensorgrams of CAR-Ig at 35 nM bound to immobilized rPenton (black, 2115 RU), fiber (gray, 889 RU), or penton base (light gray, 1061 RU). Three individual traces for each ligand are shown.

The surface exposed residues might directly contribute to the interaction of HD5 with the viral capsid. To test this hypothesis, we required a sensitive assay to measure binding. Although not precisely defined, previous studies indicated that the determinants for HD5 binding on AdV are within the vertex proteins, fiber and penton base [9], [22]. Defensin binding to the vertex stabilizes the capsid and blocks viral uncoating during cell entry [7]–[9], [23]. Accordingly, we reasoned that surface plasmon resonance (SPR) analysis of HD5 binding to penton capsomeres comprised of only fiber and penton base might circumvent a prohibitive mass difference between the intact virus (∼150 MDa) and HD5 (∼3.6 kDa). The baculovirus expression system was used to generate full-length human AdV-5 fiber that was purified by ion exchange chromatography. Human AdV-5 penton base (PB) with an N-terminal 6×His tag was created in bacterial cells and purified by cobalt affinity chromatography. Size exclusion chromatography was used to confirm the trimerization of fiber and pentamerization of PB (data not shown). To form the rPenton complex, we co-incubated fiber and PB overnight at a 2∶1 molar ratio to minimize the number of uncomplexed PB subunits in the sample. rPenton was then purified by cobalt affinity chromatography through the 6×His tag on PB. We made multiple independent rPenton preparations. In each, the presence of both PB and fiber in the final rPenton product was confirmed by immunoblot using an anti-fiber monoclonal antibody and PB anti-sera (Figure 3A). Analysis of total protein by SDS-PAGE and Coomassie stain indicated that complex formation contributed substantially to purification (Figure 3B). And, EM analysis of purified rPenton (Figure 3C) revealed features identical to intact pentons liberated from mature AdV particles by incubation under hypotonic conditions at low pH [24]. The fiber shaft, fiber knob, and PB are all clearly apparent. A few large aggregates were observed, which may be composed of PB and an unidentified non-viral contaminant (* in Figure 3B). Based on these analyses, a single rPenton preparation with the least amount of free fiber visible by EM was used for binding studies.

To evaluate the functionality of rPenton, we individually coupled the parent fiber and PB proteins as well as rPenton to the dextran matrix of serial flow cells of a CM5 chip and employed SPR to measure binding of purified CAR-Ig, a soluble form of the AdV receptor fused to the constant region of rabbit Ig. As expected, CAR-Ig was unable to bind to PB but was able to bind to fiber and rPenton (Figure 3D). Differences in the degree of binding to fiber compared to rPenton likely reflect the amount of each protein immobilized on the chip and the relative purities of the protein preparations. Taken together, these studies indicate that immobilized rPenton is a functional subunit of the AdV capsid and is a suitable substrate for binding analysis by SPR.

### HD5 binding to rPenton reflects whole virus binding

**Figure 4:**
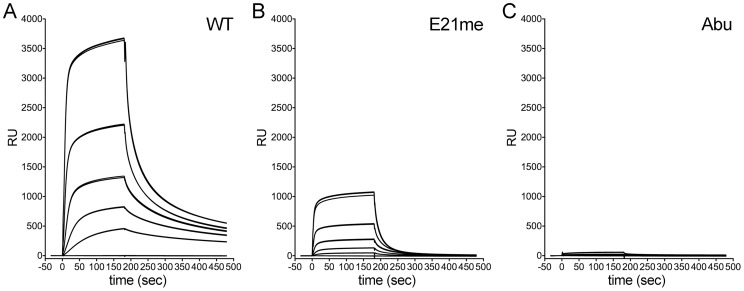
Binding of control HD5 ligands to immobilized rPenton. Background-corrected SPR sensorgrams from a concentration series of wild type HD5 (*A*), HD5 E21me (*B*), and HD5Abu (*C*). Analyte concentrations range from 9 µM to 111 nM in 3-fold dilutions, and three individual traces for each concentration are shown.

We first established conditions to measure the affinity of wild type HD5 for rPenton (Figure 4A). Immobilization of 2115 RU of rPenton yielded a response much greater than expected for 1∶1 binding. Despite prolonged injection of analyte (30 min) at 500 nM, we never achieved saturation, suggesting non-specific interaction with the dextran matrix (data not shown). This phenomenon has been previously reported and was most apparent at the highest HD5 concentration (9 µM) [25]. Nonetheless, the system was not mass transport limited in flow rate analyses (data not shown). Thus, we limited our association time to 300 sec and used a low flow rate to conserve analyte.

We then measured binding of two HD5 analogues, HD5 Abu and HD5 E21me, that were previously studied in semi-quantitative whole virus binding assays [9], [16]. HD5Abu is a linear analogue of HD5 that contains substitutions of α-aminobutyric acid (Abu) for the six cysteine residues, precluding disulfide bond formation. HD5Abu lacks anti-viral activity and does not bind to whole virus [9]. HD5 E21me cannot dimerize due to disruption of stabilizing hydrogen bonds by methylation of the peptide bond between C20 and E21 [13]. It has significantly reduced anti-viral activity, which correlates with decreased capsid binding [16]. By SPR analysis, E21me has greatly reduced binding to rPenton (Figure 4B), and HD5Abu does not bind (Figure 4C). Thus, rPenton is a reasonable proxy for whole virus.

### Stoichiometry rather than affinity dictates the anti-viral activity of HD5

**Figure 5:**
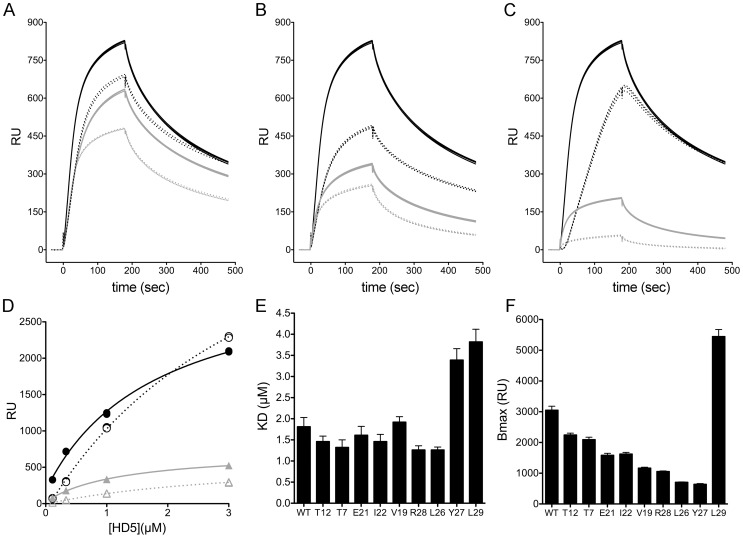
Binding of alanine scan mutants to rPenton. *A–C*, background-corrected SPR sensorgrams of defensins, each at 333 nM, binding to immobilized rPenton (2115 RU). Three individual traces for each mutant are shown. The wild type HD5 sensorgram (solid black) is identical in *A–C* for comparison with (*A*) T7A (dotted black), T12A (solid gray), and E21A (dotted gray); (*B*) I22A (dotted black), R28A (solid gray), and V19A (dotted gray); and (*C*) L29A (dotted black), L26A (solid gray), and Y27A (dotted gray). *D*, Representative steady-state binding curves for wild type HD5 (solid black), L29A (dotted black), L26A (solid gray), and Y27A (dotted gray). Dissociation constants (*E*) and maximum binding (B_max_) values (*F*) for alanine mutants. Data are best-fit values ± SE.

We selected HD5 analogues from the alanine scan with a range of inhibitory activity and analyzed their binding kinetics to rPenton by SPR. Given our results with wild type HD5, we studied a 3-fold dilution series of each analogue from 3 µM to 111 nM. The sensorgrams of 333 nM of each defensin all had similar shapes, suggesting that the on- and off-rates were comparable (Figure 5A–C). One exception was L29A (dotted black line in Figure 5C), which appeared to have a much slower on-rate. Because the sensorgrams were not well fitted to the built in analysis equations of the Biacore software and did not reflect 1∶1 binding, we derived steady-state binding curves of the data at 80 sec of association time to quantify affinity (K_D_) and maximal binding at saturation (B_max_) (Figure 5D). Wild type HD5 bound to rPenton with a K_D_ of 1.81±0.22 µM (Figure 5E). The majority of the mutants bound with similar affinity ranging from 1.26–1.92 µM. Two notable exceptions were Y27A (K_D_ = 3.39±0.27 µM) and L29A (K_D_ = 3.82±0.30 µM), which bound with much lower affinity. When ordered by their relative anti-viral activity, there was no correlation between affinity and neutralization (p = 0.57, when Y27A and L29A are excluded). In contrast, there was a positive correlation between B_max_ and anti-viral activity (p = 0.0027, when L29A is excluded). The value of B_max_ for wild type HD5 was 3050±130 RU (Figure 5F), corresponding to a stoichiometry of 210 (Equation 1). The corresponding B_max_ value for the most attenuated mutant (Y27A) was 640±25 RU. As was the case for K_D_, the behavior of L29A was unique in that it had a B_max_ higher than that of wild type HD5 (5445±230 RU). Taken together, these studies suggest that the number of HD5 molecules bound to the capsid rather than their absolute affinity dictate anti-viral activity. Nonetheless, a minimal affinity is required, since the stoichiometry of L29A exceeds that of wild type HD5 yet L29A is not capable of neutralizing infection, likely due to its lower affinity.

### Hydrophobic and charged residues are also required for anti-viral activity of the myeloid α-defensin HNP1

**Figure 6:**
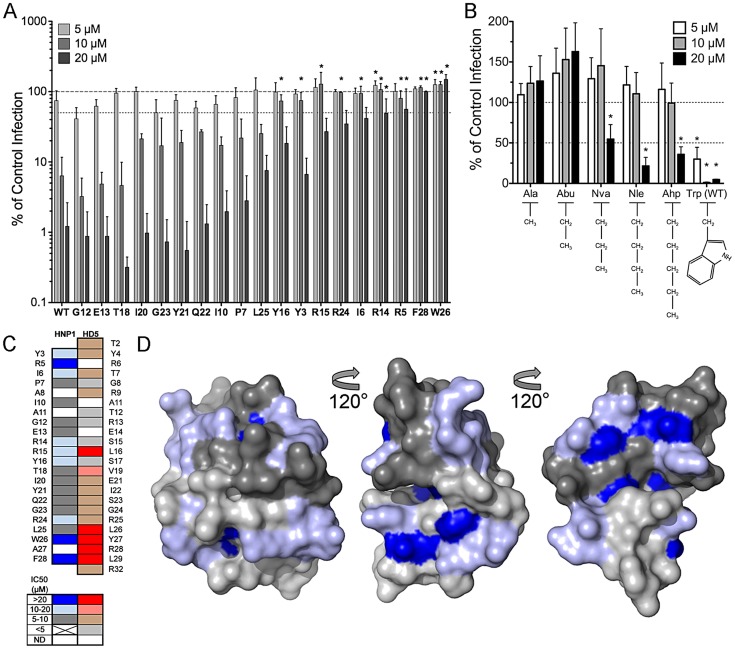
Anti-viral activity and model of HNP1 alanine scan mutants. *A*, AdV5.eGFP infection upon pre-binding of virus to A549 cells prior to addition of the indicated concentrations of α-defensins is expressed relative to control cells infected in the absence of α-defensin (100%, upper dashed line). Alanine was substituted for HNP1 residues that are indicated by position and single letter code. Lower dashed line represents 50% of control infection. Data are the means of at least three independent experiments ± SD. *, p<0.05. *B*, HNP1 analogs containing the indicated natural and non-natural amino acid substitutions at position 26 were analyzed for activity against AdV5.eGFP as in *A*. *, p<0.05 comparing each mutant to the alanine substitution. *C*, Structurally analogous residues in HD5 and HNP1 were aligned, and a heat map of the effect of alanine substitutions on their IC_50_s against AdV5.eGFP was generated according to the color key below. ND = not done. HD5 data is reproduced from Figure 2A. *D*, surface rendering of the HNP1 dimer (PDB: 3GNY) with the backbone in the same orientation as for HD5 in Figures 2B and C. Residues are colored as in *C* according to their effect on the IC_50_ of HNP1 against AdV5.eGFP. Dark and light grey are equivalent and used to distinguish the monomers comprising the dimer.

To determine whether the properties of HD5 that are required for potent anti-viral activity might extend to a second human α-defensin, we analyzed a set of alanine scan mutants of the myeloid α-defensin HNP1 for their capacity to inhibit AdV infection. Compared to HD5, HNP1 has reduced ability to neutralize AdV infection [7], [9]. In addition, we observed that HNP1 was a more potent inhibitor of AdV when added to virus already pre-bound to the cell rather than when the defensin was pre-incubated with the virus prior to addition to cells (data not shown). Consequently, we restricted our analysis to AdV, as the kinetics of HPV binding and entry preclude parallel analysis of HPV under these conditions. Approximately half of the HNP1 alanine mutants had a greater than 2-fold effect on IC_50_ (Figure 6A). Two of the most deleterious mutations were of C-terminal hydrophobic residues, W26 and F28, which align with Y27 and L29 of HD5 in linear sequence (Figure 6C). Similar to our prior studies of the contribution of hydrophobicity at position 29 of HD5 to anti-AdV activity [16], substitution of W26 with non-natural amino acids of increasing hydrophobicity partially restored the anti-viral activity of HNP1 (Figure 6B). HNP1 W26Nva had an IC_50_ of almost 20 µM, while W26Nle and W26Ahp had IC_50_s between 10 and 20 µM. Together, these data emphasize the role of hydrophobicity in α-defensin neutralization of AdV.

**Figure 7:**
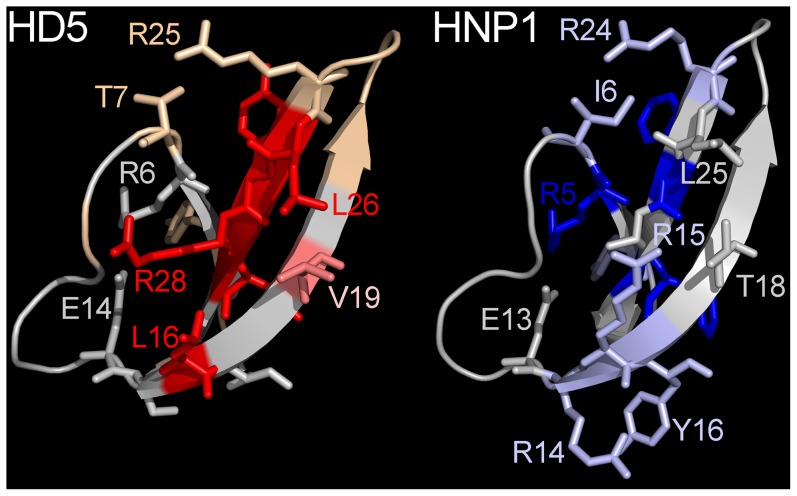
Comparison of HD5 and HNP1. Selected residues critical for anti-viral activity of HD5 (left) or HNP1 (right) against AdV are shown in stick representation and colored as in Figure 6B.

Analysis of HNP1 surface exposed residues (Figure 6D) revealed a more complex pattern than we observed for HD5 (Figure 2B). Rather than a discrete interface located on one side of the molecule, we observed two patches on opposite faces of each monomer for a total of four potential interacting surfaces on each dimer. These surfaces were mostly formed by residues that when mutated had less than a 3-fold effect on IC_50_, while for HD5 several of the prominent surface-exposed residues were the most critical. A direct comparison of the structures of HD5 and HNP1 reveals some similarities in the relative geometry of the side chains of key residues (Figure 7). For both α-defensins, the guanidinium group of an important arginine residue (R15 for HNP1 and R28 for HD5) occupies the center of one aspect of the β-sheet that comprises the α-defensin fold. Buried aromatic residues (W26 and F28 in HNP1 and Y27 and L29 in HD5) are located on the opposite side of the β-sheet and stabilize the hydrophobic core of the defensin. These residues affect the configuration of the surface exposed residues, the integrity of the dimer interface, and the ability of the defensins to self-associate and to neutralize virus infection [15], [26]. The importance of V19 in HD5 but not T18 in HNP1 underscores the functional importance of hydrophobicity; however, a surprising inconsistency was the importance of L26 in HD5 but not L25 in HNP1, which might be due to differences in their orientations. Hence, despite a lack of complete overlap in the surface-exposed residues of the two α-defensins that interact with AdV, which was expected due to their modest sequence conservation, there are common features that dictate their capacity to neutralize infection. These properties may be general to other α-defensins binding to AdV and to these α-defensins binding to other non-enveloped viruses.

## Discussion

Our previous structure-function analysis of HD5 focused on the requirement for arginine residues at specific locations in the structure as well as a crucial role for dimerization [16]. The inability of lysine to functionally substitute for arginine implied a more prominent role for properties of arginine other than mere positive charge. In addition, we identified a requirement for hydrophobicity at a key location in the defensin structure (L29) to mediate defensin-defensin interactions. Our current studies have allowed us to explore these ideas more fully and demonstrate a pronounced role for hydrophobicity in the anti-viral capacity of α-defensins. In this context, hydrophobicity can play four distinct but not mutually exclusive roles: 1) to mediate direct contacts between the virus and the defensin; 2) to stabilize the defensin dimer interface; 3) to mediate higher order defensin self-association; and 4) to serve a structural role in the hydrophobic core of the defensin that may indirectly affect the functionality of surface-exposed residues.

A subset of hydrophobic residues (L16, V19, and L26) that is important for AdV/HPV inhibition form a discrete patch on one face of the HD5 dimer. Interestingly, we found previously that a positively charged residue (R28) contiguous with these hydrophobic residues was critical for AdV neutralization but dispensable for anti-HPV activity [16]. In contrast, the negatively charged residue E21 was more important for anti-HPV activity. Unlike R28, which is more distal, E21 is centrally located at the dimer interface. These requirements likely reflect differences in the nature of the α-defensin binding site on each virus. Our understanding of these determinants is rudimentary for AdV: both fiber and penton base are necessary, yet the molecular features that dictate defensin binding have not been precisely defined [9], [22]. In contrast, corresponding HPV capsid determinants have not yet been investigated. Our studies are consistent with a model in which a central hydrophobic patch may mediate contacts with both viruses, although roles for these residues in addition to mediating direct contact cannot be excluded, while charge-charge interactions confer specificity for each virus and are dictated by residues with different charges and orientations relative to the dimer interface.

Several of the residues identified in both the HPV and AdV screens are not surface-exposed and likely do not contribute directly to virus binding. These hydrophobic residues might fulfill alternative roles such as stabilizing the dimer interface. The HD5 dimer interface is formed by interactions between S17, L29, and the C3–C31 disulfide bond of one monomer with I22 and the C5–C20 disulfide bond of the other monomer as well as reciprocal hydrogen bonds between the backbones of V19 and E21 on both monomers [13]. We previously demonstrated that mutation of L29 or disruption of backbone hydrogen bonds mediated by E21 attenuates HD5 inhibition of both AdV and HPV by disrupting dimerization [13], [16]. Whereas mutation of S17 or I22 had no effect on AdV inhibition in our current study, HPV inhibition was attenuated by mutation of I22. Thus, the HPV interaction may be even more sensitive than that of AdV to the stability of the dimer interface. This interpretation assumes that the HD5 dimer is the functional unit. I22 is surface exposed in the HD5 monomer and could be involved in recognizing determinants unique to HPV. A similar argument could be made for L29, which rather than stabilizing the dimer interface could mediate direct contact of the HD5 monomer with either virus. Unlike I22 or L29, the side chain of Y27 is buried and only minimally exposed on the surface of either the monomeric or dimeric forms of HD5. Mutation of Y27 is deleterious for both AdV and HPV inhibition. The bulky hydrophobic side chain of W26 in HNP1 adds structural rigidity to the surrounding residues [26]. Y27 may play a similar supportive role in HD5, maintaining proper orientation and functionality of the surface exposed residues. In summary, the anti-viral mechanism of HD5 against non-enveloped viruses is multifaceted and involves interplay between hydrophobicity and charge on the defensin surface to recognize viral targets as well as the need for a hydrophobic core to support dimerization and the correct orientation of the viral interface.

To test whether properties governing the potency of HD5 are general to another α-defensin, we analyzed the anti-viral activity of comparable alanine mutants of HNP1 against AdV. Like for HD5, stabilization of the dimer interface through the C-terminal hydrophobic residues W26 and F28 of HNP1 was critical. Although in linear sequence these residues are directly analogous to Y27 and L29 of HD5, respectively, their precise mode of action differs due to the distinct geometries of the dimer interface of each defensin: W26 is directly involved in HNP1 dimerization, and L29 but not Y27 mediates HD5 dimerization [13]. Accordingly, we saw some restoration of activity by substituting non-natural amino acids with increasingly hydrophobic side chains for both HNP1 W26A and HD5 L29A [16]. Mutation of W26 may have an additional effect on self-association of HNP1 independent of canonical dimer contacts [15], [26]. In further agreement with our studies of HD5, surface exposed arginine residues of HNP1 were critical. However, unlike in HD5 where there was one key arginine [16], mutation of R14, R15, and R24 attenuated HNP1 activity. Moreover, a combination of surface-exposed hydrophobic and arginine residues did not form a discrete patch on one face of HNP1. Rather, they are dispersed on multiple faces of the defensin dimer. Interestingly, disruption of the salt bridge in the R5A mutant of HNP1 led to a reduction in anti-viral activity. We had previously shown that an HD5 mutant disrupting the salt bridge through a more conservative mutation (E14Q) had no effect [9]. Since E13A, which also disrupts the salt bridge, was not attenuating in the current study, the effect of the R5A mutation must extend beyond its role in forming the salt bridge, perhaps involving the hydrophobic component of the side chain. Alternatively, a non-neutralized positive charge (in E13A) is better tolerated and may enhance function unlike a non-neutralized negative charge (in R5A). Since the equivalent residue in HD5 (R6) was not tested, we do not know if a less conservative mutation in HD5 would be similarly attenuating. Thus, some of the principles dictating HD5 anti-viral activity are predictive of qualities critical for HNP1; however, failure to identify a discrete binding interface on HNP1 may reflect its relatively lower anti-viral activity compared to HD5

The alanine scan mutagenesis also provided a means to gain insight into the mechanism of virus neutralization. An outstanding question from our previous work was the parameters of virus binding that dictate defensin potency. To address this issue, we utilized a subunit of the AdV capsid, rPenton, to correlate anti-viral activity and binding. The rationale for the use of rPenton as a surrogate for the complete capsid was based on previous studies of chimeric viruses in which substitutions of penton base and fiber dictated defensin sensitivity [9]. We found, in general, that the anti-viral efficacy of the HD5 mutants correlated most closely with their stoichiometry at saturation rather than their affinity for rPenton. For wild type HD5 and mutants with near wild type activity, there are ∼210 HD5 molecules/rPenton. Given that there are 12 pentons/virion, ∼2520 HD5 molecules would bind to the whole virus. This finding is in line with our previous semi-quantitative binding data in which we found that ∼2750 HD5 molecules bound to each complete AdV particle [9]. This high stoichiometry supports the anti-viral mechanism suggested by previous cryoEM analysis, which indicated that HD5 was binding to and coating the fiber and penton base proteins of the vertex, thereby preventing fiber dissociation [9]. These data are consistent with two models: either mutation of critical residues restricts binding of the defensins to fewer sites on the capsid or limits defensin-defensin self-association that occurs subsequent to initial defensin-capsid binding. Two exceptions to this trend were Y27A and L29A. Y27A exhibited both weaker affinity and reduced stoichiometry relative to wild type, whereas the affinity of L29A was lower but its stoichiometry was much higher than wild type. Thus, as evidenced by L29, a minimal affinity must be important for neutralization despite high stoichiometry. L29 overall exhibited a unique binding kinetic, possibly indicative of greater interaction with the dextran matrix of the chip. Alternatively, this may reflect that L29A exhibits reduced self-association [13], [25]; however, the fact that the kinetics of E21me binding had a similar shape to that of wild type argues against this unless a major contribution to binding of E21me is mediated by L29 itself. Overall, mutations that reduced anthrax lethal factor binding vs. rPenton binding differed in their relative effect, highlighting the specificity of each interaction [13]. Although SPR was a vast improvement over our semi-quantitative whole virus binding assays, some limitations include heterogeneous orientation of rPenton on the SPR chip, exposure of surfaces of rPenton that would be inaccessible to defensin binding in the intact capsid, and the presence of an impurity in the rPenton preparation that may have an influence on quantification of binding that we cannot formally exclude. In addition, a portion of the fiber protein in AdV-5 and AdV-2 is glycosylated, which is likely not recapitulated in the baculovirus-derived proteins [27]. Although glycosylation does affect the reactivity of fiber with antibodies, it has no effect on the trimerization of fiber or its incorporation into particles [28]. Moreover, most AdV serotypes that are neutralized by α-defensins are not glycosylated [9], [29]. Nonetheless, the contribution of fiber glycosylation to defensin-mediated neutralization of AdV could not be assessed in our SPR analysis. This analysis was also limited to AdV, as we have not yet identified a capsid subunit of HPV analogous to rPenton that would serve as a suitable binding partner.

In summary, this study maps for the first time a precise region on the surface of an α-defensin dimer crucial for interaction with and inhibition of non-enveloped viruses. Multiple residues involved in this interaction are distinct from those implicated in α-defensin anti-bacterial function [13]. Similar defensin interfaces have only been previously identified for HNP1 binding to Lipid II and for interaction of human β-defensin 1 with the chemokine receptor CCR6 and with *E. coli* [30], [31]. Although our comparison of HD5 and HNP1 inhibition of AdV identified some commonalities (e.g., important C-terminal hydrophobic residues) between the α-defensins, the lack of a discrete surface patch on HNP1 as well as the increased dependence on arginine residues suggests that different sets of rules dictate the anti-viral activity of each α-defensin. Further studies of α- and β-defensins are needed to define a core set of rules that govern the broad, yet selective anti-viral activity of both α- and β-defensins, which may allow for the development of novel therapeutics based on defensin mechanisms.

## Materials and Methods

### Cells, viruses, and peptides

Tissue culture reagents were obtained from Mediatech (Manassas, VA) or Invitrogen (Carlsbad, CA). Human A549, HeLa, 293β5, and 293TT cells were cultured in DMEM supplemented with 10% fetal bovine serum (Sigma-Aldrich, St. Louis, MO), 4 mM L-glutamine, 100 units/ml penicillin, 100 µg/ml streptomycin, and 0.1 mM nonessential amino acids (complete DMEM) as previously described [16].

The replication-defective human AdV-5 vector used in these studies (AdV5.eGFP) is E1/E3-deleted and contains an enhanced green fluorescent protein (eGFP) reporter gene cassette driven by a CMV promoter. AdV5.eGFP was propagated in 293β5 cells, purified by CsCl gradient centrifugation, stored and used as previously described [8], [16]. HPV16 pseudoviruses (PsVs) containing L1 and L2 were produced in 293TT cells and purified according to established protocols [16], [32], [33].

Synthetic HD5 and HNP1 were obtained from Peptides International, Inc. (Louisville, KY). Alternatively, folded HD5 was generated from a synthesized 80% pure linear peptide (CPC Scientific, Sunnyvale, CA) by thiol-disulfide reshuffling and purified to homogeneity by reverse-phase high-pressure liquid chromatography [16], [34]. The synthesis, refolding, purification, and structural validation of the HD5 and HNP1 alanine scan mutants have been described [13], [26]. All α-defensins were quantified by UV absorbance at 280 nm using calculated molar extinction coefficients [35].

### Quantification of virus infection

A549 cell monolayers were infected with serial dilutions of AdV5.eGFP in black wall, clear bottom 96-well plates (Perkin-Elmer, San Jose, CA). Total monolayer fluorescence was quantified with a Typhoon 9400 variable mode imager (GE Healthcare, Piscataway, NJ) 24–30 h post-infection. A virus concentration producing 50–80% maximal signal was chosen for inhibition studies.

Serum-free DMEM (SFM) alone or containing increasing concentrations of wild type or mutant HD5 was incubated with purified AdV5.eGFP for 45 min on ice. The mixture (35 µl/well) was added to a confluent monolayer of A549 cells that had been washed twice in SFM. Cells were then incubated at 37°C for 2 h with rocking, washed, and cultured with complete DMEM for 24–30 h. Plates were scanned for eGFP signal as above, and background-subtracted total well fluorescence was quantified using ImageJ software [36]. Experiments with HPV PsVs were performed as above with the following exceptions: 1) infection was measured on HeLa cells, 2) the PsVs were incubated at 37°C for 4 h prior to washing and removal of the inoculum, and 3) GFP was measured 48 h post-infection. For assays with HNP1, virus alone in SFM was pre-incubated on a confluent monolayer of A549 cells with rocking for 45 min at 4°C. Cells were then washed twice with chilled SFM, and SFM alone or containing increasing concentrations of wild type or mutant HNP1 was added. Cells were incubated for 45 min at 4°C and then shifted to 37°C. After 2 h, cells were washed twice with complete media and incubated a further 24–30 h at 37°C until eGFP signal was quantified as above.

### Purification of human AdV5 fiber

An N-terminal 6×His tag followed by a Tev protease recognition site was cloned 5′ of the full-length human AdV5 fiber gene into pFastBAC. Recombinant baculovirus was made in Sf21 cells using the Bac-to-Bac system (Invitrogen) following the manufacturer's instructions. Protein expression was in HighFive cells in shaker flasks (135 rpm, 27°C, 3 days) at a multiplicity of infection between 1 and 3. Cell pellets were frozen at −80°C in lysis buffer (50 mM Na_2_HPO_4_/NaH_2_PO_4_, 50 mM NaCl, 0.1% Triton X-100, pH 8.0), thawed, and lysed by sonication in the presence of Halt EDTA-free protease inhibitors (Thermo Fisher Scientific). Initial purification of clarified lysate with TALON (Clontech) resin indicated that the 6×His tag was lost from the majority of the recombinant fiber. Thus, the flow through from the TALON column was purified by FPLC on a Q Sepharose Fast Flow column (GE Healthcare) using a linear gradient from 0 to 300 mM NaCl in 25 mM Tris pH 7.4. Fractions containing fiber were pooled and concentrated. Fiber trimerization was confirmed by immunoblot of samples heated or not for 5 min to 95°C using the 4D2 anti-fiber mAb (Thermo Fisher Scientific) and by analytical size exclusion chromatography using a Superdex 200 10/300 GL column (GE Healthcare)(data not shown).

### Purification of human AdV5 penton base

A bacterial expression plasmid (pRSET-A) encoding an N-terminal 6×His tag followed by an enterokinase recognition site 5′ of the full-length human AdV5 penton base (PB) gene was a gift from Lali Medina-Kauwe [37]. PB was expressed in BL21-CodonPlus (DE3)-RIPL cells (Stratagene) upon induction at an OD_600_ of 0.6–0.8 using 0.4 mM IPTG for 4 hr at 37°C. Cell pellets were frozen at −80°C in lysis buffer. Thawed cells were lysed by sonication in the presence of 1 mM PMSF, treated with 0.01 mg/ml DNase I for 10 min at RT, adjusted to 300 mM NaCl and 10 mM imidazole, and clarified by centrifugation (18,000× g for 45 min). Clarified lysate was applied to a TALON column and washed sequentially with 10 column volumes each of MCAC-10 (50 mM Na_2_HPO_4_/NaH_2_PO_4_, 300 mM NaCl, 0.1% Triton X-100, 10 mM imidazole, pH 8.0) and MCAC-20 (50 mM Na_2_HPO_4_/NaH_2_PO_4_, 500 mM NaCl, 20 mM imidazole, 10% glycerol, pH 8.0). Bound protein was eluted with MCAC-250 (50 mM Na_2_HPO_4_/NaH_2_PO_4_, 300 mM NaCl, 250 mM imidazole, 10% glycerol, pH 8.0). Fractions containing the highest concentrations of eluted protein were pooled and concentrated/desalted into desalting buffer (50 mM Na_2_HPO_4_/NaH_2_PO_4_, 130 mM NaCl, 10% glycerol, pH 8.0). PB oligomerization was confirmed by analytical size exclusion chromatography as for fiber above (data not shown).

### Purification of rPenton

Concentrated PB (∼2.9 µM) and fiber (∼5.8 µM) were mixed in the presence of Halt EDTA-free protease inhibitors and incubated overnight at 4°C in a total volume of 800 µl. rPenton was then purified on TALON beads as for PB above except that the wash and elution buffers contained 150 mM NaCl. Peak fractions were pooled, concentrated, and stored in desalting buffer. Fractions from purification were resolved by SDS-PAGE. Immunoblots were probed for fiber (4D2) and PB (rabbit antiserum, a kind gift from Glen R. Nemerow) and visualized with chemiluminescence.

### Electron microscopy

Purified rPenton was diluted to 13 µg/ml in PBS. Samples were negatively stained with 2% uranyl acetate on 400 mesh carbon coated grids (Ted Pella, Inc.) that were glow discharged for 30 s at 15 microamps and imaged on a FEI Tecnai TF20 Transmission electron microscope at 200 kV and a nominal magnification of 60,000× at the Cleveland Center for Membrane and Structural Biology.

### CAR-Ig purification

A gene encoding residues 1 to 235 of the human coxsackie and adenovirus receptor (CAR) was cloned in frame with the Fc portion of the heavy chain of rabbit IgG in the expression plasmid pCB6 [38]. This construct was transfected into 293 cells, and a polycolonal population of cells (293-CAR-Ig) was selected with G418 (0.5 mg/ml, Cellgro). Bovine Ig was depleted from FBS using the Affi-Gel Protein A MAPS II Kit (Bio-rad). 293-CAR-Ig cells were cultured in DMEM/4% Ig-depleted FBS for 48 h, and CAR-Ig was purified from the culture supernatant using the Affi-Gel Protein A MAPS II Kit following the manufacturer's instructions, concentrated, and stored in 25 mM Tris, 150 mM NaCl, pH 7.0.

### Surface plasmon resonance (SPR)

Experiments were performed on a Biacore T200 system (Biacore, GE Healthcare) at 25°C in HBS EP+ running buffer (9.5 mM HEPES, 142.5 mM NaCl, 2.85 mM EDTA, 0.05% surfactant P20, pH 7.4). The first flow cell on a CM5 sensor chip was reserved to measure background binding, while additional cells were used to immobilize fiber, PB, and rPenton by amine-coupling chemistry. A target density of 2000 resonance units (RU) was selected for rPenton, and for Figure 3D, an equal number of fiber (708 RU) and PB (1292 RU) were also targeted for immobilization. The final RUs immobilized were: 2115 RU rPenton, 889 RU fiber, and 1061 RU PB. Analyte was injected to the flow cells at a rate of 11 µl/min and binding was measured for 3 min, followed by measuring dissociation for 5 min in analyte-free running buffer. The flow cells were regenerated with two 30 s pulses of 10 mM HCl at 11 µl/min separated by 30 s in running buffer. After regeneration, the flow cells were stabilized in running buffer for 2 min before injection of the next analyte. Data analysis was performed using Biacore T200 evaluation software and Prism (version 5.0d).

### Stoichiometry

Affinity (K_D_) and B_max_ were derived by fitting RU values at 80 s post-injection using the Steady State Surface Bound Affinity model in Biacore T200 Evaluation software. Stoichiometry (Sm) of HD5 bound to rPenton at saturation was calculated using the following equation: 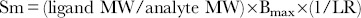
(1)


Ligand response (LR) is the amount of immobilized ligand in RU.

### Statistical analysis

Experiments were analyzed using Prism 5.0d. For Figures 1A, 1B, and 6A, data were analyzed by two-way ANOVA with Bonferroni post-tests to compare each mutant to wild type HD5 or HNP1 at each concentration. For Figure 6B the post-tests were used to compare each mutant or wild type to HPN1 W26A. Pearson correlation analyses between K_D_ and IC_50_ or between B_max_ and IC_50_ were performed using IC_50_ values calculated from the data in Figure 1 and binding data from Figure 5. Values for Y27 and L29 (K_D_) or only for L29 (B_max_) were excluded from the correlation analyses. For all tests, p<0.05 was considered significant.
